# Phenotypic and Molecular Characterization of Antimicrobial Resistance in *Klebsiella* spp. Isolates from Companion Animals in Japan: Clonal Dissemination of Multidrug-Resistant Extended-Spectrum β-Lactamase-Producing *Klebsiella pneumoniae*

**DOI:** 10.3389/fmicb.2016.01021

**Published:** 2016-06-29

**Authors:** Kazuki Harada, Takae Shimizu, Yujiro Mukai, Ken Kuwajima, Tomomi Sato, Masaru Usui, Yutaka Tamura, Yui Kimura, Tadashi Miyamoto, Yuzo Tsuyuki, Asami Ohki, Yasushi Kataoka

**Affiliations:** ^1^Department of Veterinary Internal Medicine, Tottori UniversityTottori, Japan; ^2^Laboratory of Veterinary Microbiology, Nippon Veterinary and Life Science UniversityTokyo, Japan; ^3^Laboratory of Food Microbiology and Food Safety, Rakuno Gakuen UniversityHokkaido, Japan; ^4^Miyamoto Animal HospitalYamaguchi, Japan; ^5^Sanritsu Zelkova Veterinary LaboratoryKanagawa, Japan; ^6^Monoris Co., Ltd.Tokyo, Japan

**Keywords:** *Klebsiella* spp., dogs, cats, extended-spectrum β-lactamases, multidrug resistance, clonal dissemination

## Abstract

The emergence of antimicrobial resistance in *Klebsiella* spp., including resistance to extended-spectrum cephalosporins (ESC) and fluoroquinolones, is of great concern in both human and veterinary medicine. In this study, we investigated the prevalence of antimicrobial resistance in a total of 103 *Klebsiella* spp. isolates, consisting of *Klebsiella pneumoniae* complex (KP, *n* = 89) and *K. oxytoca* (KO, *n* = 14) from clinical specimens of dogs and cats in Japan. Furthermore, we characterized the resistance mechanisms, including extended-spectrum β-lactamase (ESBL), plasmid-mediated AmpC β-lactamase (PABL), and plasmid-mediated quinolone resistance (PMQR); and assessed genetic relatedness of ESC-resistant *Klebsiella* spp. strains by multilocus sequence typing (MLST) and pulsed-field gel electrophoresis (PFGE). Antimicrobial susceptibility testing demonstrated that resistance rates to ampicillin, cephalothin, enrofloxacin, ciprofloxacin, trimethoprim/sulfamethoxazole, cefotaxime, gentamicin, tetracycline, chloramphenicol, amoxicillin-clavulanic acid, and cefmetazole were 98.1, 37.9, 37.9, 35.9, 35.0, 34.0, 31.1, 30.1, 28.2, 14.6, and 6.8%, respectively. Phenotypic testing detected ESBLs and/or AmpC β-lactamases in 31 of 89 (34.8%) KP isolates, but not in KO isolates. Resistances to 5 of the 12 antimicrobials tested, as well as the three PMQRs [*qnrB, qnrS*, and *aac(6*′*)-Ib-cr*], were detected significantly more frequently in ESBL-producing KP, than in non-ESBL-producing KP and KO. The most frequent ESBL was CTX-M-15 (*n* = 13), followed by CTX-M-14 (*n* = 7), CTX-M-55 (*n* = 6), SHV-2 (*n* = 5), CTX-M-2 (*n* = 2), and CTX-M-3 (*n* = 2). Based on the *rpoB* phylogeny, all ESBL-producing strains were identified as *K. pneumoniae*, except for one CTX-M-14-producing strain, which was identified as *K. quasipneumoniae*. All of AmpC β-lactamase positive isolates (*n* = 6) harbored DHA-1, one of the PABLs. Based on MLST and PFGE analysis, ST15 KP clones producing CTX-M-2, CTX-M-15, CTX-M-55, and/or SHV-2, as well as KP clones of ST1844-CTX-M-55, ST655-CTX-M-14, and ST307-CTX-M-15, were detected in one or several hospitals. Surprisingly, specific clones were detected in different patients at an interval of many months. These results suggest that multidrug-resistant ESBL-producing KP were clonally disseminated among companion animals via not only direct but also indirect transmission. This is the first report on large-scale monitoring of antimicrobial-resistant *Klebsiella* spp. isolates from companion animals in Japan.

## Introduction

Members of the genus *Klebsiella*, belonging to the Enterobacteriaceae, are gram-negative bacilli that inhabit freshwater environments including surface water, sewage and soil, as well as the mucosal surfaces of mammals (Podschun and Ullmann, 1998). *Klebsiella pneumoniae* is the most medically-important species in the genus and is responsible, together with *K. oxytoca* (KO), for nosocomial infections in humans (Podschun and Ullmann, 1998). Strains classically identified as *K. pneumoniae* have been previously subdivided into phylogroups named KpI, KpII, and KpIII (Brisse et al., 2014). Recently, it has been proposed that these phylogroups be redesignated as distinct species, *K. pneumoniae* (KpI), *K. quasipneumoniae* (KpII), and *K. variicola* (KpIII) (Holt et al., 2015), which are collectively called *K. pneumoniae* complex (KP). In companion animals, *Klebsiella* spp. have been demonstrated to cause infections such as urinary tract infections (Ling et al., 2001), pyometra (Stone et al., 1988), upper respiratory infections (Adler et al., 2007), and septicemia (Roberts et al., 2000).

The emergence of antimicrobial resistance in *Klebsiella* spp. isolates is of great concern worldwide in human medicine (Lynch et al., 2013). It increases the risk of antimicrobial treatment failure not only in humans but also in companion animals. Similarly, the emergence of antimicrobial-resistant bacteria in companion animals may have important human public health consequences if isolates are transmitted to humans by their pets (Guardabassi et al., 2004; Lloyd, 2007). Understanding the prevalence of antimicrobial resistance among *Klebsiella* spp. isolates is thus important both from veterinary medicine and public health perspectives.

Resistance to extended-spectrum cephalosporins (ESC) and fluoroquinolones in gram-negative bacteria, including *Klebsiella* spp., is of particular concern (Paterson, 2006). ESC resistance is mainly associated with the production of plasmid-mediated extended-spectrum β-lactamases (ESBLs), AmpC β-lactamases (PABLs), and carbapenemases (Kenneth, 2010; Rubin and Pitout, 2014). Although fluoroquinolone resistance is mainly acquired by modification of their target enzymes, it may also involve acquisition of plasmid-mediated quinolone resistance (PMQR) determinants (Fàbrega et al., 2005; Guillard et al., 2015). PMQRs determine relatively small increases in quinolone resistance, but these changes are sufficient to mediate natural selection of mutants that have higher levels of resistance (Strahilevitz et al., 2009). In recent years, these important resistance mechanisms in *Klebsiella* spp. isolates from companion animals have been well documented in several European countries, including Germany (Stolle et al., 2013; Ewers et al., 2014), Italy (Donati et al., 2014), France (Haenni et al., 2012; Poirel et al., 2013), Spain (Hidalgo et al., 2013), and Switzerland (Wohlwend et al., 2015). However, the status of emerging antimicrobial resistance in *Klebsiella* spp. in companion animals remains unknown in many other countries, including Japan.

The aim of the present study was to investigate the prevalence of antimicrobial resistance, and provide molecular characterization of ESC resistance and PMQRs in *Klebsiella* spp. isolates from clinical specimens from dogs and cats visited to different veterinary hospitals throughout Japan. A further aim was to assess the epidemiological relatedness of ESC-resistant *Klebsiella* spp. strains.

## Materials and methods

### Bacterial isolates

A total of 103 *Klebsiella* spp. isolates, consisting of 89 KP and 14 KO, were obtained from clinical specimens collected from dogs (*n* = 78) and cats (*n* = 25) that visited veterinary hospitals between 2003 and 2015. These hospitals were located at the following 15 prefectures in Japan: Hokkaido, Fukui, Gunma, Ibaraki, Saitama, Tokyo, Chiba, Kanagawa, Nagano, Aichi, Osaka, Hyogo, Tottori, Yamaguchi, and Fukuoka prefectures. The specimens were isolated from various anatomical sites, assessed as being sites of bacterial infection by clinical veterinarians, including the urinary tract (*n* = 48), skin (*n* = 11), genitals (*n* = 9), and respiratory organs (*n* = 9), ears (*n* = 7), and digestive organs (*n* = 6); as well as pus from unspecified locations (*n* = 8), and body fluids including ascites (*n* = 4). One specimen was of unknown anatomical origin. The details of *Klebsiella* spp. isolates used in this study are shown in Supplementary Table 1. No information was available regarding previous antimicrobial treatment of the dogs and cats. This study was carried out in accordance with the recommendations of Guidelines for Proper Conduct of Animal Experiments, Science Council of Japan. All enrolled animals received the best practice veterinary care, and the owners granted informed consent. Bacterial identification was conducted by growth status on CHROMagar orientation medium (Ohkusu, 2000), using the API 20E kit (SYSMEX bioMérieux Co., Ltd., Tokyo, Japan). Because KP and KO often present similar biochemical patterns (Chander et al., 2011), the species-specific PCR was further carried out for confirmation of bacterial species, as previously reported (Chander et al., 2011). All confirmed *Klebsiella* spp. isolates were stored at −80°C in 10% skim milk.

### Antimicrobial susceptibility testing

Susceptibilities to ampicillin (AMP), amoxicillin-clavulanic acid (ACV), cephalothin (CPL), cefmetazole (CMZ), cefotaxime (CTX), meropenem (MPM), tetracycline (TET), gentamicin (GEN), chloramphenicol (CHL), trimethoprim/sulfamethoxazole (TMS), ciprofloxacin (CIP), and enrofloxacin (ENR) were determined. Susceptibility testing was conducted using the agar dilution method, according to the Clinical and Laboratory Standards Institute (CLSI) guidelines (CLSI, 2013a). The results obtained were interpreted as per the criteria contained within CLSI guidelines (CLSI, 2013b,c). *Escherichia coli* ATCC 25922 was used as a control strain.

ESC-resistant (i.e., CTX MIC ≥ 2 μg/mL) strains were screened for production of ESBLs and AmpC-type β-lactamases using a commercial combination disk kit (MAST Diagnostics, Co. Ltd., UK), which can separately detect the two type of β-lactamases by comparing the inhibition zones of the cefpodoxime (CPD) disk to the inhibition zones of each of the CPD plus inhibitor (i.e., ESBL inhibitor, AmpC inhibitor, or both) disks.

### Detection of PMQR genes

Genomic DNA from each of the isolates was prepared by suspending several colonies in 0.5 ml of water and boiling for 10 min. These samples were used as templates for further genetic analyses. All isolates were screened for eight PMQR genes [i.e., *qnrA, qnrB, qnrC, qnrD, qnrS, qepA, aac(6*′*)-Ib-cr*, and *oqxAB* genes] using multiplex PCR (Ciesielczuk et al., 2013). Positive genes were confirmed subsequently by single PCR. Randomly selected PCR products of *oqxAB* and all of the products of the other PMQRs were directionally sequenced with the same primers, for confirmation.

### Characterization of β-lactamase genes in ESC-resistant *Klebsiella* spp. strains

All of the ESC-resistant strains were screened for class A β-lactamase genes (i.e., *bla*TEM and *bla*SHV), which were identified using PCR and DNA sequencing, as previously reported (Kojima et al., 2005). In the isolates with ambiguous sequences of *bla*SHV, indicating that they have both chromosomal and plasmid *bla*SHV genes (Haanperä et al., 2008), the two *bla*SHV genes were separately identified, as previously described (Lee et al., 2006). In ESBL-positive strains, the CTX-M-type β-lactamase genes were detected using multiplex PCR (Xu et al., 2007); for the positive isolates, the genes were amplified and sequenced to identify CTX-M subtypes using group-specific PCR primers (Kojima et al., 2005; Shibata et al., 2006). In AmpC-positive strains, PABL genes (i.e., ACC, FOX, MOX, DHA, CIT, and EBC groups) were screened by multiplex PCR (Pérez-Pérez and Hanson, 2002), amplified and then bi-directionally sequenced using specific primers (Yan et al., 2002).

### Multilocus sequence typing and phylogenetic analysis of ESC-resistant *Klebsiella* spp. strains

For ESC-resistant KP strains, multilocus sequence typing (MLST) with seven genes (i.e., *gapA, infB, mdh, pgi, phoE, rpoB*, and *tonB*) was carried out according to the protocol on the Institut Pasteur website (http://bigsdb.web.pasteur.fr/klebsiella/klebsiella.html). New alleles and STs were submitted to the MLST website and new ST numbers were assigned. eBURST v3 analysis (http://eburst.mlst.net/v3/instructions/) was performed to identify clonal complexes (CCs), defined as groups of two or more independent isolates sharing identical alleles at six or more loci (Ewers et al., 2014). Following Breurec et al. (2013), we further split the STs into clonal groups (CGs) mapping within the large central CC.

In addition, the phylogenetic relationship of all ESC-resistant strains was investigated based on the *rpoB* gene sequence (Brisse et al., 2014).

### Pulsed-field gel electrophoresis of ESBL-producing *Klebsiella* spp. strains

Pulsed-field gel electrophoresis (PFGE) was performed on ESBL-producing KP (ESBL-KP) strains, as previously described (Herschleb et al., 2007). DNA embedded in agarose was digested with *Xba*I (Takara Bio, Inc., Japan) and then electrophoresed using CHEF DRIII (Bio-Rad, Hercules, CA, USA). PFGE profiles were digitized for analysis using BioNumerics software (version 5.10; Applied Maths, TX, USA). All fragment sizes within the gel were normalized using the molecular weight method. A similarity matrix was calculated using the Dice coefficient, and cluster analysis was performed using the UPGMA algorithm. A cluster was defined based on a similarity cut-off of 80% with 1.0% optimization and 1.0% band tolerance.

### Statistical analysis

Prevalence of antimicrobial resistance and PMQR genes between the three groups (i.e., ESBL-KP and non-ESBL-KP, and KO strains) was compared using the Fisher's exact test. A *P* value lower than 0.05 was considered significant. The Bonferroni correction for multiple comparisons was applied, lowering the threshold for significance to a value of *P* ≤ 0.0167.

## Results

### Antimicrobial susceptibilities of *Klebsiella* spp. isolates

The numbers of isolates with resistance to AMP, CPL, ENR, CIP, TMS, CTX, GEN, TET, CHL, ACV, and CMZ were 101 (98.1%), 39 (37.9%), 39 (37.9%), 37 (35.9%), 36 (35.0%), 35 (34.0%), 32 (31.1%), 31 (30.1%), 29 (28.2%), 15 (14.6%), and 7 (6.8%), respectively, in 103 *Klebsiella* spp. clinical isolates (Supplementary Table 1). All of the isolates tested had low MIC for MEM (≤0.031 to 0.063 μg/mL).

Based on MIC of CTX, ESC resistance was detected in 35 KP isolates but not in KO isolates. The combination disk screening test revealed that 31 of 35 (88.6%) ESC-resistant KP strains produced ESBLs with or without AmpC β-lactamases, whereas the remaining four isolates produced AmpC β-lactamases but not ESBLs. Resistances against TET, GEN, TMS, CIP, and ENR, as well as cephalosporins including CPL and CTX, were detected significantly more frequently in ESBL-KP strains, than in non-ESBL-KP and KO strains (*P* < 0.0167, Table 1).

**Table 1:** **MIC distribution and resistance rates among *Klebsiella* spp. isolates from dogs and cats in Japan**.

**Antimicrobials^a^**	**Species^b^**	**No. of isolates**	**MIC (μg/ml)^c^**															**No. of resistant isolates (%)^d^**
			**≤ 0.031**	**0.063**	**0.125**	**0.25**	**0.5**	**1**	**2**	**4**	**8**	**16**	**32**	**64**	**128**	**256**	**>256**	
AMP	KP (ESBL−)	58								1		1	5	18	21	3	9	56 (96.6)
	KP (ESBL+)	31															31	31 (100)
	KO	14												2	9	2	1	14 (100)
ACV	KP (ESBL−)	58							20	22	8		3	5				8 (13.8)
	KP (ESBL+)	31									10	14	5	2				7 (22.6)
	KO	14							4	8		2						0 (0)
CPL	KP (ESBL−)	58							5	19	22	6		1		1	4	6 (10.3)
	KP (ESBL+)	31															31	31 (100)^*^
	KO	14							2	3	3	4	1				1	2 (14.3)
CMZ	KP (ESBL−)	58					3	32	9	7	2			2	3			5 (8.6)
	KP (ESBL+)	31						5	18	4	1		1		2			2 (6.5)
	KO	14					6	3	2	2			1					0 (0)
CTX	KP (ESBL−)	58	3	26	18	6	1			1		3						4 (6.9)
	KP (ESBL+)	31									1		2	3	5	7	13	31 (100)^*^
	KO	14	2	5	3	3		1										0 (0)
MPM	KP (ESBL−)	58	14	44														0 (0)
	KP (ESBL+)	31	5	26														0 (0)
	KO	14	11	3														0 (0)
TET	KP (ESBL−)	58						29	10	8	1			2	2	4	2	10 (17.2)
	KP (ESBL+)	31						8	3			1		1	1	9	8	20 (64.5)^*^
	KO	14				2	7	1		1	2	1						1 (7.1)
GEN	KP (ESBL−)	58				7	38	6					1	2	2		2	7 (12.1)
	KP (ESBL+)	31					6					1		12	9	3		25 (80.6)^*^
	KO	14				2	9	3										0 (0)
CHL	KP (ESBL−)	58							1	14	24	4	5	2	1	4	3	15 (25.9)
	KP (ESBL+)	31							1	4	16			2			8	10 (32.3)
	KO	14							4	5		1	4					4 (28.6)
CIP	KP (ESBL−)	58	11	28	2	2		4	3	1	1		1	2		3		8 (13.8)
	KP (ESBL+)	31						1	1		2	11	9	5	1	1		29 (93.5)^*^
	KO	14	9	1	2	1			1									0 (0)
ENR	KP (ESBL−)	58	2	17	15	8	1	4	2	1		2	2		2	2		9 (15.5)
	KP (ESBL+)	31						1	1	1	4	7	11	4	2			29 (93.5)^*^
	KO	14	2	7	1	3				1								1 (7.1)
						≤0.25/4.75			0.5/9.5	1/19	2/38	4/76	8/152	16/304	32/608	64/1216	>64/1216	
TMS	KP (ESBL−)	58						41	1	4	3	2					7	9 (15.5)
	KP (ESBL+)	31						1	2	1					1		26	27 (87.1)^*^
	KO	14						13	1									0 (0)

a AMP, ampicillin; ACV, amoxicillin-clavulanic acid; CPL, cephalothin; CMZ, cefmetazole; CTX, cefotaxime; MPM, meropenem; TET, tetracycline; GEN, gentamicin; CHL, chloramphenicol; CIP, ciprofloxacin; ENR, enrofloxacin; TMS, trimethoprim/sulfamethoxazole.

b P, K. pneumoniae complex; KO, K. oxytoca.

c Vertical lines indicate the breakpoint for each drug, according to CLSI guidelines (Atasoy et al., 2012; Bales et al., 2011).

d Significantly higher resistance rates than non-ESBL-producing KP and KO isolates (^*^ P < 0.0167).

### Prevalence of PMQR genes in *Klebsiella* spp. isolates

Of the eight PMQRs tested, *qnrB, qnrS, aac(6*′*)-Ib-cr*, and *oqxAB* genes were detected in KP clinical isolates, whereas none of the PMQR genes were detected in KO isolates (Table 2). The *qnrB, qnrS*, and *aac(6*′*)-Ib-cr* genes were significantly more prevalent in ESBL-KP strains than in non-ESBL-KP strains (respectively 29.0 vs. 5.2, 25.8 vs. 3.4, and 35.5 vs. 1.7%, *P* < 0.0167). However, the *oqxAB* gene was detected in approximately 90% of isolates of both ESBL-KP and non-ESBL-KP strains: there was no significant difference in prevalence of the gene between the strains (*P* > 0.05).

**Table 2:** **Prevalence of eight PMQRs among *Klebsiella* spp. isolates from dogs and cats in Japan**.

**PMQR genes**	***K. pneumoniae*** **complex**		***K. oxytoca* (*n* = 14)**
	**ESBL-negative (*n* = 58)**	**ESBL-positive (*n* = 31)**	
*qnrA*	0 (0)	0 (0)	0 (0)
*qnrB*	3 (5.2)	9 (29.0)^a^	0 (0)
*qnrC*	0 (0)	0 (0)	0 (0)
*qnrD*	0 (0)	0 (0)	0 (0)
*qnrS*	2 (3.4)	8 (25.8)^a^	0 (0)
*qepA*	0 (0)	0 (0)	0 (0)
*aac(6′)-Ib-cr*	1 (1.7)	11 (35.5)^a^	0 (0)
*oqxAB*	53 (91.4)^b^	28 (90.3)^b^	0 (0)

a Significantly higher resistance rates than non-ESBL-producing K. pneumoniae complex and K. oxytoca isolates (P < 0.0167).

b Significantly higher resistance rates than K. oxytoca isolates (P < 0.0167).

### Prevalence of β-lactamases among ESC-resistant *Klebsiella* spp. strains

Table 3 shows the detailed characteristics of 35 ESC-resistant clinical strains. Twenty-seven and four ESC-resistant strains harbored one and two ESBL genes, respectively. Of the ESBL genes detected in this study, CTX-M-15 was the most prevalent (*n* = 13), followed by CTX-M-14 (*n* = 7), CTX-M-55 (*n* = 6), SHV-2 (*n* = 5), CTX-M-2 (*n* = 2), and CTX-M-3 (*n* = 2). All of the six AmpC-positive strains (*n* = 6), two of which were also positive for ESBLs, harbored DHA-1, one of the PABLs. As for β-lactamases other than ESBLs and PABLs, SHV-1 and TEM-1 were prevalent (*n* = 24 and 13, respectively); other minor β-lactamases including SHV-11, SHV-26, SHV-27, OKP-B, and TEM-176 were also detected (*n* = 4, 2, 2, 1, and 1, respectively). Three ESC-resistant strains harbored two types of SHV genes (i.e., SHV-1 and SHV-2).

**Table 3:** **Characterization of 35 ESC-resistant *Klebsiella* spp. strains from dogs and cats in Japan**.

**Isolate**	**Animals**	**Origin**	**MLST**		**Phenotype**	**ESBLs and/or AmpC**	**Other β-lactamases**	**PMQR**	**Resistance to antimicrobials^c^**
			**ST**	**CC/CG**					
***K. pneumoniae***									
KL19	Dog	Urine	15	15^b^	ESBL	CTX-M-15	SHV-1	*oqxAB*	AMP-CPL-CTX-GEN-SXT-CIP-ENR
KL21	Cat	Urine	15		ESBL	CTX-M-15	SHV-1	*oqxAB*	AMP-CPL-CTX-GEN-SXT-CIP-ENR
KL22	Cat	Urine	15		ESBL	CTX-M-15	SHV-1	*oqxAB*	AMP-CPL-CTX-GEN-SXT-CIP-ENR
KL23	Cat	Urine	15		ESBL	CTX-M-15	SHV-1	*oqxAB*	AMP-CPL-CTX-GEN-SXT-CIP-ENR
KL25	Cat	Stomach	15		ESBL	CTX-M-15	SHV-1	*oqxAB*	AMP-CPL-CTX-GEN-SXT-CIP-ENR
KL35	Cat	Urine	15		ESBL	CTX-M-15	SHV-1	*oqxAB*	AMP-CPL-CTX-GEN-SXT-CIP-ENR
KL38	Dog	Urine	15		ESBL	CTX-M-55, SHV-2	-	*aac(6′)-Ib-cr, oqxAB*	AMP-CPL-CTX-GEN-SXT-CIP-ENR
KL51	Dog	Lung	15		ESBL+AmpC	SHV-2, DHA-1	TEM-1, SHV-1	*qnrB, oqxAB*	AMP-ACV-CPL-CMZ-CTX-TET-GEN-CIP-ENR
KL54	Dog	Urine	15		ESBL	CTX-M-2	SHV-1	*oqxAB*	AMP-CPL-CTX-TET-CHL-GEN-CIP-ENR
KL61	Dog	Blood	15		ESBL	CTX-M-55	SHV-1	*qnrB, aac(6′)-Ib-cr, oqxAB*	AMP-CPL-CTX-TET-GEN-SXT-CIP-ENR
KL94	Cat	Urine	15		ESBL	CTX-M-15	SHV-1	*oqxAB*	AMP-CPL-CTX-GEN-SXT-CIP-ENR
KL96	Cat	Urine	15		ESBL	CTX-M-55, SHV-2	TEM-1, SHV-1	*aac(6′)-Ib-cr*	AMP-CPL-CTX-TET-GEN-SXT-CIP-ENR
KL105	Cat	Urine	15		ESBL	SHV-2	SHV-1	*aac(6′)-Ib-cr, oqxAB*	AMP-CPL-CTX-TET-CHL-GEN-SXT-CIP-ENR
KL108	Dog	Pus	15		ESBL	CTX-M-15	TEM-1, SHV-1	*oqxAB*	AMP-CPL-CTX-TET-GEN-CIP-ENR
KL114	Cat	Nasal cavity	15		ESBL	CTX-M-55, SHV-2	TEM-1	*qnrB, aac(6′)-Ib-cr, oqxAB*	AMP-CPL-CTX-TET-GEN-SXT-CIP-ENR
KL120	Cat	Urine	15		ESBL	CTX-M-15	SHV-1	*oqxAB*	AMP-CPL-CTX-GEN-SXT-CIP-ENR
KL24	Cat	Pleural effusion	655	15^b^	AmpC	DHA-1	SHV-1	*qnrB, oqxAB*	AMP-ACV-CPL-CMZ-CTX-GEN-SXT-CIP-ENR
KL40	Dog	Genitals	655		ESBL	CTX-M-14	SHV-1	*qnrS, oqxAB*	AMP-ACV-CPL-CTX-TET-CHL-GEN-SXT-CIP-ENR
KL74	Dog	Nasal cavity	655		ESBL+AmpC	CTX-M-14, DHA-1	TEM-1, SHV-1	*qnrB, qnrS, oqxAB*	AMP-ACV-CPL-CMZ-CTX-TET-CHL-GEN-SXT-CIP-ENR
KL99	Dog	Urine	655		ESBL	CTX-M-14	SHV-1	*qnrS, oqxAB*	AMP-CPL-CTX-TET-GEN-SXT-CIP-ENR
KL109	Cat	Skin	655		ESBL	CTX-M-14	SHV-1	*qnrS, oqxAB*	AMP-CPL-CTX-TET-GEN-SXT-CIP-ENR
KL26	Dog	Urine	1844^a^	15^b^	ESBL	CTX-M-55	SHV-1	*oqxAB*	AMP-CPL-CTX-TET-CHL-SXT-CIP-ENR
KL27	Dog	Urine	1844^a^		ESBL	CTX-M-55	SHV-1	*oqxAB*	AMP-CPL-CTX-SXT-CIP-ENR
KL39	Dog	Oral cavity	37	37^b^	AmpC	DHA-1	TEM-1, SHV-11	*aac(6′)-Ib-cr, oqxAB*	AMP-ACV-CPL-CMZ-CTX-TET-CHL-GEN-SXT-CIP-ENR
KL60	Dog	Urine	709	515^b^	ESBL	CTX-M-2, CTX-M-15	TEM-1, SHV-26	*oqxAB*	AMP-CPL-CTX-TET-CHL-CIP-ENR
KL59	Cat	Skin	337	37	ESBL	CTX-M-14	SHV-11	*qnrB, qnrS, aac(6′)-Ib-cr, oqxAB*	AMP-ACV-CPL-CTX-TET-CHL-SXT-CIP-ENR
KL53	Dog	Urine	881	37	ESBL	CTX-M-14	TEM-1, SHV-27	*qnrS, oqxAB*	AMP-CPL-CTX-TET-CHL-GEN-SXT
KL57	Cat	Urine	307	307	ESBL	CTX-M-15	TEM-1, SHV-1	*qnrB, aac(6′)-Ib-cr, oqxAB*	AMP-ACV-CPL-CTX-TET-GEN-SXT-CIP-ENR
KL103	Dog	Urine	307		ESBL	CTX-M-15	TEM-1, SHV-1	*aac(6′)-Ib-cr, oqxAB*	AMP-CPL-CTX-SXT-CIP-ENR
KL104	Cat	Pus	307		ESBL	CTX-M-15	TEM-1, SHV-1	*qnrB, aac(6′)-Ib-cr, oqxAB*	AMP-ACV-CPL-CTX-TET-GEN-SXT-CIP-ENR
KL111	Dog	Urine	34	34	ESBL	CTX-M-3	SHV-26	*qnrB, aac(6′)-Ib-cr, oqxAB*	AMP-CPL-CTX-TET-SXT-CIP
KL58	Dog	Urine	147	147	ESBL	CTX-M-3	TEM-1, SHV-11	*qnrB, aac(6′)-Ib-cr, oqxAB*	AMP-ACV-CPL-CTX-TET-CHL-GEN-SXT-CIP-ENR
KL36	Dog	Ascites fluid	753	–	AmpC	DHA-1	SHV-27	*qnrB, oqxAB*	AMP-ACV-CPL-CMZ-CTX-GEN
KL118	Dog	Urine	2173^a^	–	AmpC	DHA-1	TEM-176, SHV-11	*qnrS, oqxAB*	AMP-ACV-CPL-CMZ-CTX-TET-GEN-SXT-ENR
***K. quasipneumoniae***									
KL33	Dog	Skin	–	–	ESBL	CTX-M-14	TEM-1, OKP-B	*qnrS*	AMP-CPL-CTX-TET-CHL-GEN-SXT-ENR

a New ST.

b CGs within CC37.

c AMP, ampicillin; ACV, amoxicillin-clavulanic acid; CPL, cephalothin; CMZ, cefmetazole; CTX, cefotaxime; MPM, meropenem; TET, tetracycline; GEN, gentamicin; CHL, chloramphenicol; CIP, ciprofloxacin; ENR, enrofloxacin; TMS, trimethoprim/sulfamethoxazole.

### Phylogenetic relationship of ESC-resistant *Klebsiella* spp. strains

The *rpoB* sequences of 35 ESC-resistant KP clinical strains were compared with those of seven type strains of *Klebsiella* genus (Supplementary Figure 1). The sequence of *rpoB* allele 1, which was identified in 29 strains, differed from those of the three type strains (*K. pneumoniae* subsp. *pneumoniae* ATCC 13883, *K. pneumoniae* subsp. *ozaenae* ATCC 11296, and *K. pneumoniae* sub sp. *rhinoscleromatis* CIP52-210) by 1 nucleotide (99.8% similarity). The sequence of *rpoB* allele 4, which was identified in four strains, was identical with those of *K. pneumoniae* subsp. *pneumoniae* ATCC 13883 and *K. pneumoniae* subsp. *ozaenae* ATCC 11296, whereas the *rpoB* sequence of the KL111 strain (allele 7) differed from these two subspecies by 1 nucleotide (99.8% similarity). The *rpoB* sequence of the KL33 strain (allele 13) was identical with that of *K. quasipneumoniae* subsp. *similipneumoniae* 07A044T.

As the result of phylogenetic analysis, all ESC-resistant strains fell within the *K. pneumoniae* cluster (KpI), except for the KL33 strain, which fell within the *K. quasipneumoniae* cluster (KpII).

### MLST typing of ESC-resistant *Klebsiella* spp. strains

**Figure 1:**
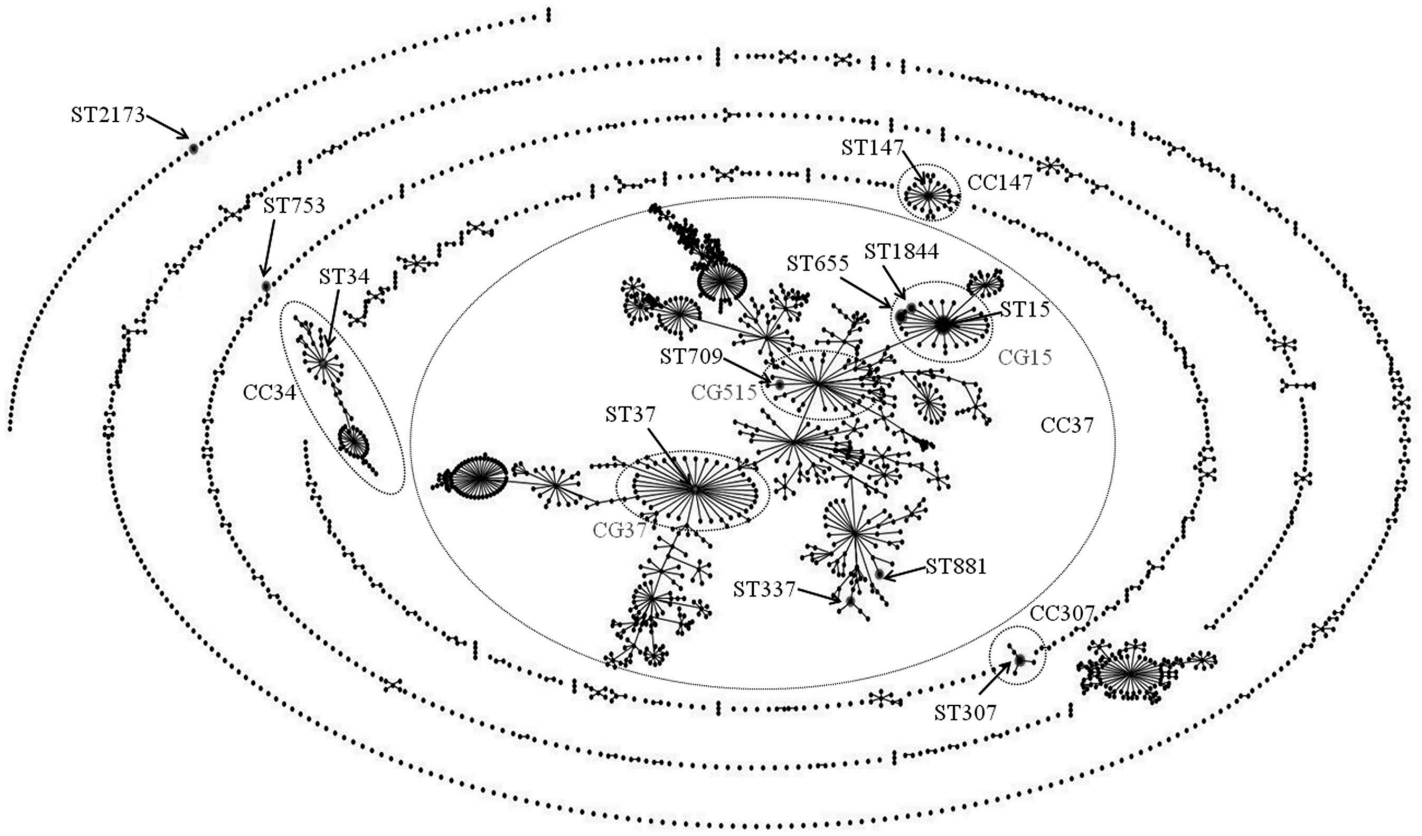
**Population snapshot by eBURST analysis for ESC-resistant *K. pneumoniae* strains against the entire *K. pneumoniae* MLST database**. The STs identified in this study are labeled with arrows. The names of the CCs are based on the ST assigned as the founder genotype. The relative size of the circles indicates the prevalence of STs and lines between STs connect SLVs. The straggly CC in the center of the picture was further separated into CGs, characterized as subsets of this complex (Breurec et al., 2013).

As shown in Table 3, 34 ESC-resistant *K. pneumoniae* clinical isolates investigated by MLST were assigned to 13 Sequence Types (STs): ST15 (allelic profile 1-1-1-1-1-1-1, *n* = 16), ST655 (1-1-1-1-1-1-23, *n* = 5), ST307 (4-1-2-52-1-1-7, *n* = 3), ST1844 (1-1-1-1-1-1-297, *n* = 2), ST34 (2-3-6-1-9-7-4, *n* = 1), ST37 (2-9-2-1-13-1-16, *n* = 1), ST147 (3-4-6-1-7-4-38, *n* = 1), ST337 (2-1-11-1-1-1-13, *n* = 1), ST709 (1-1-1-1-1-1-4, *n* = 1), ST753 (14-1-2-1-135-4-12, *n* = 1), ST881 (2-68-1-1-10-4-13, *n* = 1), and ST2173 (4-6-19-13-1-4-22, *n* = 1). Figure 1 illustrates a population snapshot by eBURST analysis for our collection, against 2250 previously-reported STs obtained from the MLST database (http://bigsdb.pasteur.fr/klebsiella/klebsiella.html: accessed on 4 February 2016). Seven of the thirteen STs generated in our collection were placed into CC37. In this CC, ST15 and its single locus variants (SLVs, ST655 and ST1844) were included in CG15; ST37 and ST709 were included in CG37 and CG515, respectively. Of non-CC37 STs, ST34, ST147, and ST307 were defined as predicted founders of their respective CCs.

### PFGE analysis of ESBL-producing *Klebsiella* spp. strains

**Figure 2:**
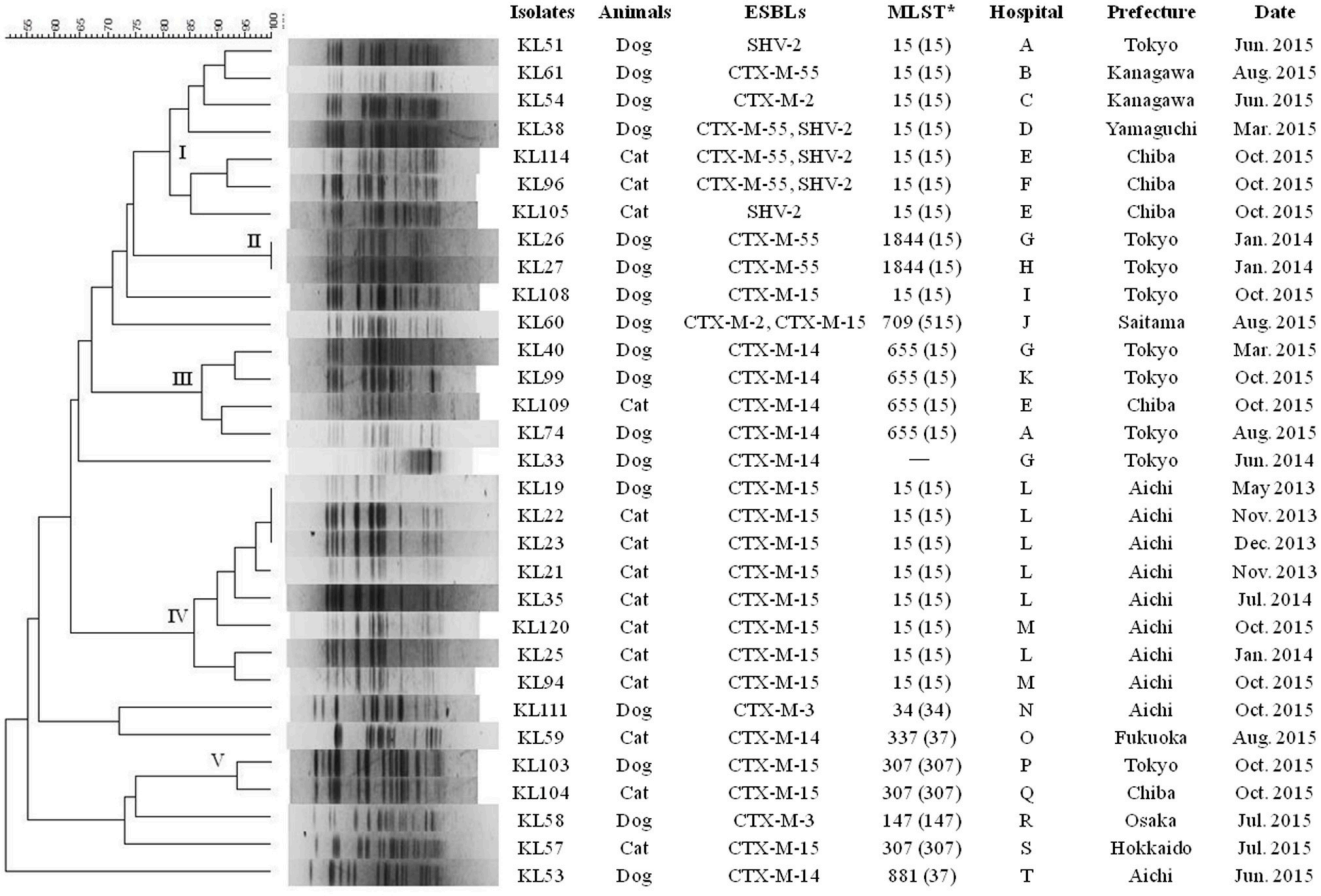
**PFGE profiles of 31 ESBL-producing *Klebsiella* spp. strains from dogs and cats in Japan**. The numbers embedded in the phylogenetic tree mean clusters. ^*^The CG or CC of each ST is given in parenthesis. ST was not assigned to KL33 strain because this strain was identified as *K. quasipneumoniae*.

In the PFGE analysis, ESBL-KP clinical strains formed five distinct clusters (Figure 2). Cluster I consisted of ST15 strains producing CTX-M-2, CTX-M-55, and/or SHV-2 obtained from the same or different veterinary hospitals in Tokyo and the neighboring prefectures (Supplementary Figure 2), except for the KL38 strain. Clusters II and III contained two ST1844-CTX-M-55 strains and four ST655-CTX-M-15 strains, respectively, obtained from several hospitals located mainly in Tokyo prefecture. Cluster IV consisted of eight ST15-CTX-M-15 strains obtained from two hospitals located in Aichi prefecture. Cluster V contained two ST307-CTX-M-15 strains obtained from different prefectures. Clusters I, III, and IV each contained strains detected at an interval of several months. The remaining strains were independent of the clusters.

## Discussion

There have been few reports on the prevalence of antimicrobial resistance in overall populations of *Klebsiella* spp. clinical isolates from companion animals, although numerous investigations focusing on the strains with resistance to cephalosporins and/or fluoroquinolones have been carried out. This study demonstrated that more than 30% of *Klebsiella* spp. isolates in our collection exhibited resistance to CPL, CTX, TET, GEN, TMS, CIP, or ENR, in addition to extremely high prevalence of AMP resistance, possibly due to chromosomal β-lactamases (Hæggman et al., 2004). On the other hand, all of the *Klebsiella* spp. isolates exhibited low MICs against MEM (≤0.063 μg/ml), which were much lower than the screening cut-offs for the detection of carbapenemases proposed by both CLSI (2013c) and European Committee on Antimicrobial Susceptibility Testing (Hrabák et al., 2014). The resistance rates in our collection were higher than those of KP as human respiratory pathogen in Japan, in which resistance rates against 35 of 36 tested antimicrobials were less than 10% (Yanagihara et al., 2015). Likewise, lower resistance rates (i.e., less than 20% in most tested antimicrobials) were found in 17 German canine and feline *Klebsiella* spp. isolates (Grobbel et al., 2007). We have previously found similar inter-country differences in prevalence of antimicrobial resistance in other pathogens from companion animals (Harada et al., 2012a,b, 2014). Systematic inter-country evaluation of *Klebsiella* spp. isolates from companion animals would provide a better understanding of country-specific trends in antimicrobial resistance.

We found higher prevalence of ESBLs in KP clinical isolates (31/89, 34.8%), compared with those from companion animals in Italy (15/70, 21.4%, Donati et al., 2014), and in Germany and other European countries (84/1112, 7.6%, Ewers et al., 2014). In addition, this carriage rate of ESBLs was extremely higher than that in human isolates in Japan (8/484, 1.6%, Sato et al., 2015). These data suggest that the risk of ESBL carriage is relatively high in KP clinical isolates from companion animals in Japan. In this study, ESBL-KP strains frequently exhibited resistance against not only cephalosporins but also the other classes of antimicrobials. This finding indicates a strong inclination toward multidrug resistance in ESBL-KP, similar to KP human isolates (Hyle et al., 2005). This high prevalence of multidrug resistance may contribute to the selection and persistence of ESBL-KP in clinical settings (Coque et al., 2008), and should be taken into account when treating companion animals with KP infections. Genetic analysis of β-lactamases revealed extremely high prevalence of CTX-M-type β-lactamases, especially CTX-M-15, in ESC-resistant KP clinical strains, similar to previous studies (Donati et al., 2014; Ewers et al., 2014). CTX-M-3 was also identified in this study, which has been reported in KO isolates in France (Ewers et al., 2014). As for ESBLs detected in KP human strains in Japan, CTX-M-14 was predominant, whereas CTX-M-15 was in the minority (Nagasaka et al., 2015). To the best of our knowledge, CTX-M-2, CTX-M-14, and CTX-M-55 were first detected in *Klebsiella* spp. isolates from companion animals.

As for β-lactamases other than ESBLs, all of the identified AmpC-type β-lactamases were DHA-1 in ESC-resistant KP clinical strains, in accordance with previous reports of KP isolates from humans (Nagasaka et al., 2015). Likewise, Wohlwend et al. (2015) have reported that DHA-1 is mainly responsible for ESC resistance in KP isolates from companion animals in a veterinary hospital in Switzerland. SHV-type β-lactamases, including one kind of ESBL (i.e., SHV-2), were detected in nearly all of the ESC-resistant KP strains. Furthermore, OKP-type β-lactamase, closely related to SHV-type, was firstly identified in *Klebsiella* spp. isolates from companion animals. The OKP-type enzymes have been specifically found in the phylogenetic group KpII of *K. pneumoniae* (Hæggman et al., 2004; Fevre et al., 2005), namely, *K. quasipneumoniae*. In fact, the OKP-B-positive strain (KL33) was identified as *K. quasipneumoniae* based on the *rpoB* phylogeny. It might be necessary to investigate the significance of *K. quasipneumoniae* as pathogen and antimicrobial resistance reservoirs in companion animals in the future.

The MLST analysis revealed that ST15 was the most common among ESBL-KP clinical strains from companion animals in Japan. In particular, ST15 clone was identified more frequently in cats than in dogs, in contrast to the other STs, indicating the spread of ST15 clone among cats has a role in its prevalence. Previous studies in Italy and France have found that ST101 and ST274 clones were predominant in ESBL-KP from companion animals (Poirel et al., 2013; Donati et al., 2014). These data indicate that the predominant ST in ESBL-KP varies by country. In this study, we found predominance of ST15-CTX-M-15, a well-known international clone of KP (Ewers et al., 2014), which was also detected in France (Haenni et al., 2012) and Germany (Ewers et al., 2014). Based on the PFGE analysis, this clone was disseminated among different patients visiting the same hospital (cluster IV). This result strongly suggests nosocomial infections of ESBL-KP clone, and similar findings have previously been reported (Haenni et al., 2012; Poirel et al., 2013; Ewers et al., 2014). The PFGE analysis also identified the other ST15 clone (cluster I). This clone consisted of strains that produce various ESBLs and were obtained from several hospitals. Thus, we identified not only intra- but also inter-hospital dissemination of ST15 ESBL-KP clones among companion animals. In addition to ST15 clone, we found that several ESBL-KP clones are spread among different hospitals, most of which were located in the same or neighboring prefecture. Surprisingly, several clones were repeatedly identified at an interval of many months, implying that ESBL-KP clones can be maintained inside or outside of hospitals for a long period. Therefore, the dissemination of ESBL-KP clones among companion animals may occur not only via direct spread from animal to animal, but also via indirect transmission from potential reservoirs and sources in the environment, as often seen in human medicine (Hendrik et al., 2015). Our data emphasizes the need for infection control in hospitals and in the community to prevent dissemination of ESBL-KP clones among companion animals.

Nagasaka et al. (2015) carried out MLST analysis for ESC-resistant KP strains from human patients in Japan. They reported a high prevalence of CG11, CG17, and CG37 with a minority of the other STs. In our collection, however, CG15, an international clonal group (Breurec et al., 2013), was predominant. On other hand, we confirmed the presence of several STs in common with human isolates (i.e., ST37 and ST147) as a minor population. These findings suggest that transmission of ESC-resistant KP between companion animals and humans may occur but is relatively uncommon in Japan. Poirel et al. (2013) have shown that CTX-M-15 genes in KP isolates from companion animals were located on a different plasmid from that of human isolates, in France, and indicated that these KP isolates evolved separately from the human reservoir. In contrast, the German study by Ewers et al. (2014) indicated that ST15-CTX-M-15 KP strains were shared by humans and animals. Hidalgo et al. (2013) firstly identified IncR plasmids, which have often been associated with human isolates, in KP isolates from dogs and cats. Further studies would be needed to clarify whether animals can act as a reservoir of antimicrobial-resistant KP, including ESBL-KP, for humans.

Of the detected PMQRs in this study, *oqxAB* is detected in nearly all of KP clinical isolates but not in KO isolates. This result can be explained by the fact that the PMQR is a chromosomally-encoded gene in KP (Yuan et al., 2012). As for other PMQRs, *aac(6*′*)-Ib-cr, qnrB*, and *qnrS* were relatively prevalent in our collection, and similar findings were confirmed in ESBL-KP isolates from human in Japan (Nagasaka et al., 2015). In contrast, the latter two PMQRs have hardly been detected in ESBL-KP isolates from companion animals in European countries (Donati et al., 2014; Ewers et al., 2014). These findings may suggest local spread of PMQRs among companion animals and humans in Japan. Furthermore, these PMQRs were more prevalent in ESBL-KP strains than non-ESBL-KP and KO isolates. This implies an epidemiological link between genes of PMQRs and ESBLs (Cantón and Coque, 2006; Strahilevitz et al., 2009). Such a link may explain the higher rates of fluoroquinolone resistance in ESBL-KP clinical strains, compared with non-ESBL-KP strains, as seen in this study.

In conclusion, we carried out the first large-scale monitoring of antimicrobial resistance in *Klebsiella* spp. clinical isolates from companion animals in Japan. Our data demonstrated a high prevalence of multidrug-resistant ESBL-KP strains, most of which harbored PMQRs. In addition, ESBL-KP strains were predominantly identified as to the ST15-CTX-M-15 clone, and scarcely contained human-related ST clone. Epidemiological data may suggest that ESBL-KP isolates are disseminated clonally, via intrahospital and interhospital transmission. Overall, *Klebsiella* spp. isolates in our collection exhibited higher rates of antimicrobial resistance and ESBL carriage than human clinical isolates in Japan, but the similarity of STs and ESBL types between isolates from human and companion animals were less common. We strongly believe that ESBL-KP poses a serious threat of antimicrobial resistance to companion animal medicine, especially in Japan, although the public health risk of ESBL-KP isolates from companion animals remains to be assessed.

## Author contributions

KH designed and coordinated the study, performed genetic analysis including detection of resistance genes and MLST analysis, and wrote the draft of manuscript. KH, TS, YM, and KK carried out the identification and antimicrobial susceptibility testing of *Klebsiella* spp. TS and MU conducted PFGE analysis. YK (MAH), TM, YT, AO, and YK (NVLU) participated in the collection of *Klebsiella* spp. isolates. All authors read and approved the final manuscript.

## Funding

This work was financially supported by JSPS KAKENHI Grant Number 24780299.

### Conflict of interest statement

The authors declare that the research was conducted in the absence of any commercial or financial relationships that could be construed as a potential conflict of interest.

## Supplementary material

Supplementary Table 1 **The details of *Klebsiella spp*. isolates used in this study**. ^a^AMP, ampicillin; ACV, amoxicillin-clavulanic acid; CPL, cephalothin; CMZ, cefmetazole; CTX, cefotaxime; MPM, meropenem; TET, tetracycline; GEN, gentamicin; CHL, chloramphenicol; TMS, trimethoprim/sulfamethoxazole; CIP, ciprofloxacin; ENR, enrofloxacin. R, resistant.

Supplementary Figure 1 **Phylogenetic relationship based on the *rpoB* gene sequence**. The tree was obtained with the neighbor-joining method with Kimura's two-parameter distance. Strain name is indicated for each sequence. The values at the nodes correspond to the bootstrap values obtained after 1000 replicates (Brisse et al., 2014).

Supplementary Figure 2 **Geographical distribution of 31 ESBL-producing *Klebsiella* spp. strains detected in this study in Japan**. Bold type denotes prefecture names. The numbers of strains are shown in parentheses.
