# Supplementary figures and images for: Phenotypic and Molecular Characterization of Antimicrobial Resistance in Klebsiella spp. Isolates from Companion Animals in Japan: Clonal Dissemination of Multidrug-Resistant Extended-Spectrum β-Lactamase-Producing Klebsiella pneumoniae
Source: Front Microbiol. 2016 Jun 29;7:1021. doi: 10.3389/fmicb.2016.01021 (PMC4925667; doi:10.3389/fmicb.2016.01021)

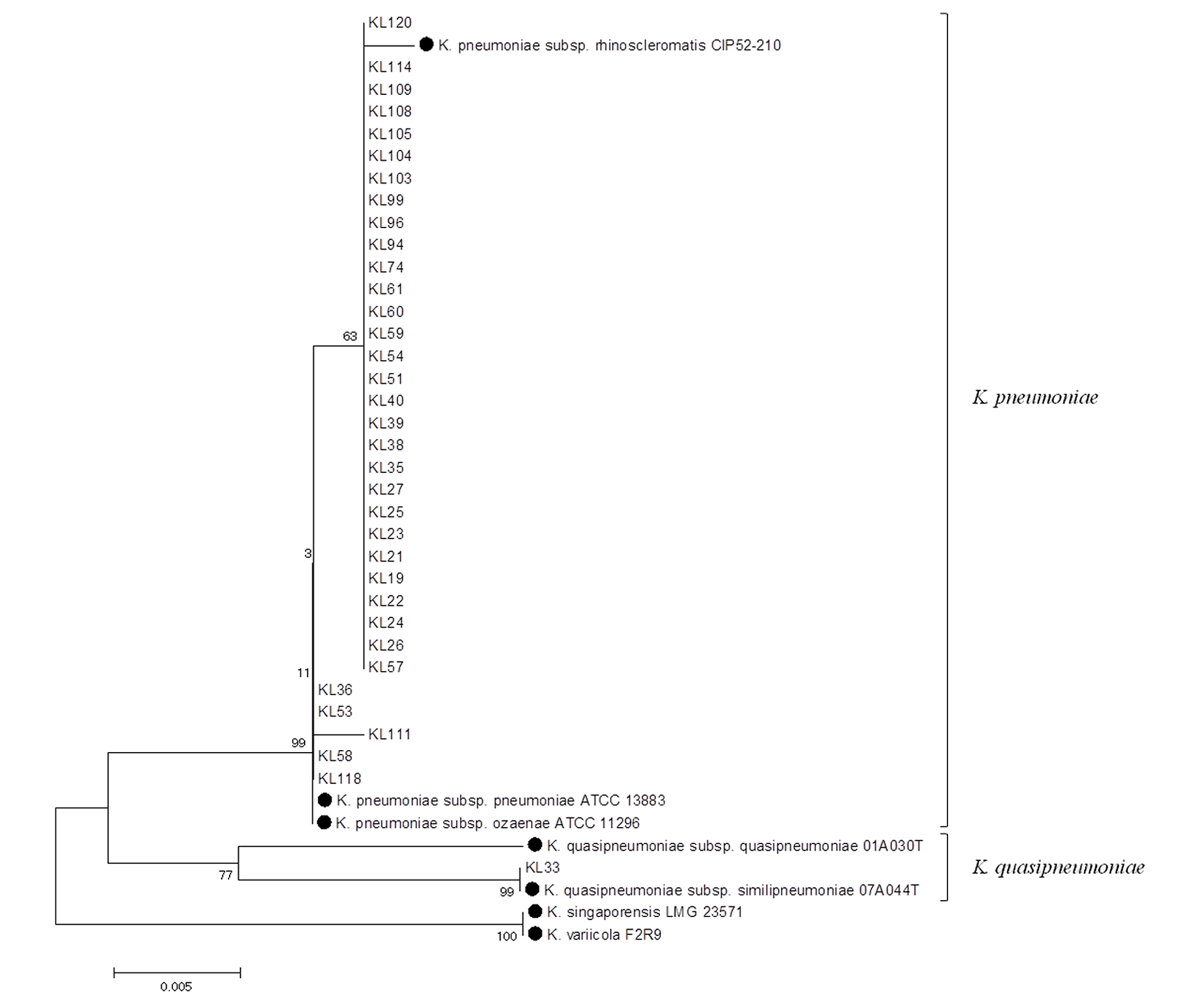

Supplement: Supplementary Figure 1 — Phylogenetic relationship based on the rpoB gene sequence. The tree was obtained with the neighbor-joining method with Kimura's two-parameter distance. Strain name is indicated for each sequence. The values at the nodes correspond to the bootstrap values obtained after 1000 replicates (Brisse et al., 2014). [file Image1.TIF]

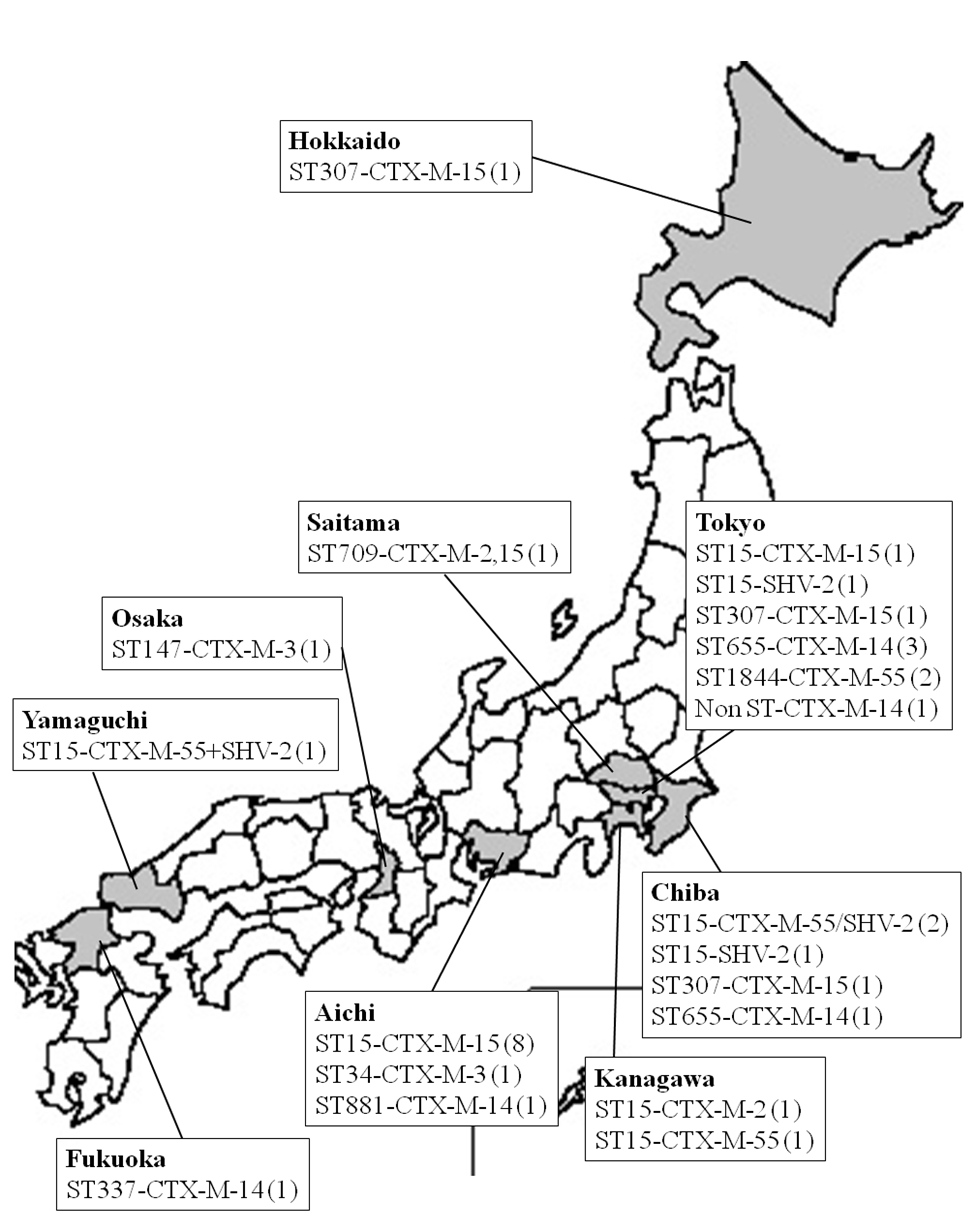

Supplement: Supplementary Figure 2 — Geographical distribution of 31 ESBL-producing Klebsiella spp. strains detected in this study in Japan. Bold type denotes prefecture names. The numbers of strains are shown in parentheses. [file Image2.TIF]
